# Site‐Specific Growth and Printing of Nanowires for Resource Efficient Fabrication of Flexible Electronics

**DOI:** 10.1002/smll.202412685

**Published:** 2025-03-17

**Authors:** D. Shakthivel, A. Christou, F. Liu, R. Dahiya

**Affiliations:** ^1^ Bendable Electronics and Sustainable Technologies (BEST) Group Electrical and Computer Engineering Department Northeastern University Boston MA 02115 USA

**Keywords:** contact printing, patterned growth, printed electronics, UV sensors, ZnO nanowires

## Abstract

Semiconducting nanowires (NWs) hold great potential for high‐performance flexible electronics. However, using them, to fabricate electronic devices, is a complex process requiring multiple lithography steps to address the issues such as the one arising from mismatches between the temperatures needed for NW growth and the temperatures the polymeric substrates can withstand. Herein, a facile “design to fab” approach is presented, which avoids lithography‐based fabrication by implementing the device layout at the NW synthesis level itself. This is demonstrated by synthesizing the arrays of ZnO NWs at pre‐defined locations, followed by their direct printing on flexible substrates using custom contact printing method. The ZnO NWs‐based printed nanoscale electronic layers exhibit excellent spatial uniformity (NW length, 18–26 µm) and alignment (88–96°). The patterned electronic layers are further processed (e.g., printed conductive tracks) at room temperature to develop proof of concept UV photodetectors. The presented approach significantly reduces the fabrication complexity by eliminating the lithography‐related steps and lays the foundation for resource‐efficient fabrication of NWs‐based large‐area flexible electronics.

## Graphical abstract

Semiconducting nanowires (NWs) hold great potential for high‐performance flexible electronics. This work demonstrates a lithography‐free approach for flexible electronics, synthesizing ZnO nanowire arrays directly at pre‐defined locations, followed by seamless dry contact printing onto rigid and flexible substrates. This “design to fab” approach simplifies fabrication, eliminating lithography and enabling resource‐efficient, large‐area flexible electronics.

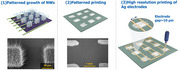

## 1 Introduction

Large‐area, flexible electronics has enabled various innovative applications including electronic skin, wearable systems, energy generation and storage devices, and bendable displays.^[^ ^1^, ^2^, ^3^, ^4^ ^]^ This has been fueled by the use of novel nanomaterials and their processing to develop various types of devices on flexible substrates.^[^ ^5^, ^6^ ^]^ Among the wide variety of nanomaterials, the inorganic 1D nanowires (NWs) (e.g., Si, Ge, GaAs, ZnO, CNTs, In_2_O_3_ etc.) are particularly attractive due to features such as single crystallinity, tunable electronic properties, chemical stability, and mechanical flexibility, which make them ideal for various electronic and sensing applications.^[^ ^7^, ^8^, ^9^, ^10^ ^]^ Several NW alignment and printing techniques such as field assisted dielectrophoresis (DEP),^[^ ^11^ ^]^ bubble‐blown alignment,^[^ ^12^ ^]^ the Langmuir‐Blodgett technique,^[^ ^13^ ^]^ and contact/transfer printing^[^ ^14^ ^]^ etc. have been studied in the past to develop flexible and large area sensors and devices. However, most of the current protocols used to develop sensors and other electronics devices from them, rely heavily on resource intensive lithography and etching fabrication steps, which create enormous toxic by‐products (e.g., photoresists/developers, acid wastes, etc.).^[^ ^15^ ^]^ Further, the compatibility of these methods with many polymeric substrates is not guaranteed and therefore an alternative manufacturing method, that can address the above issues while preserving similar device performance, is highly desirable.

**Figure 1:**
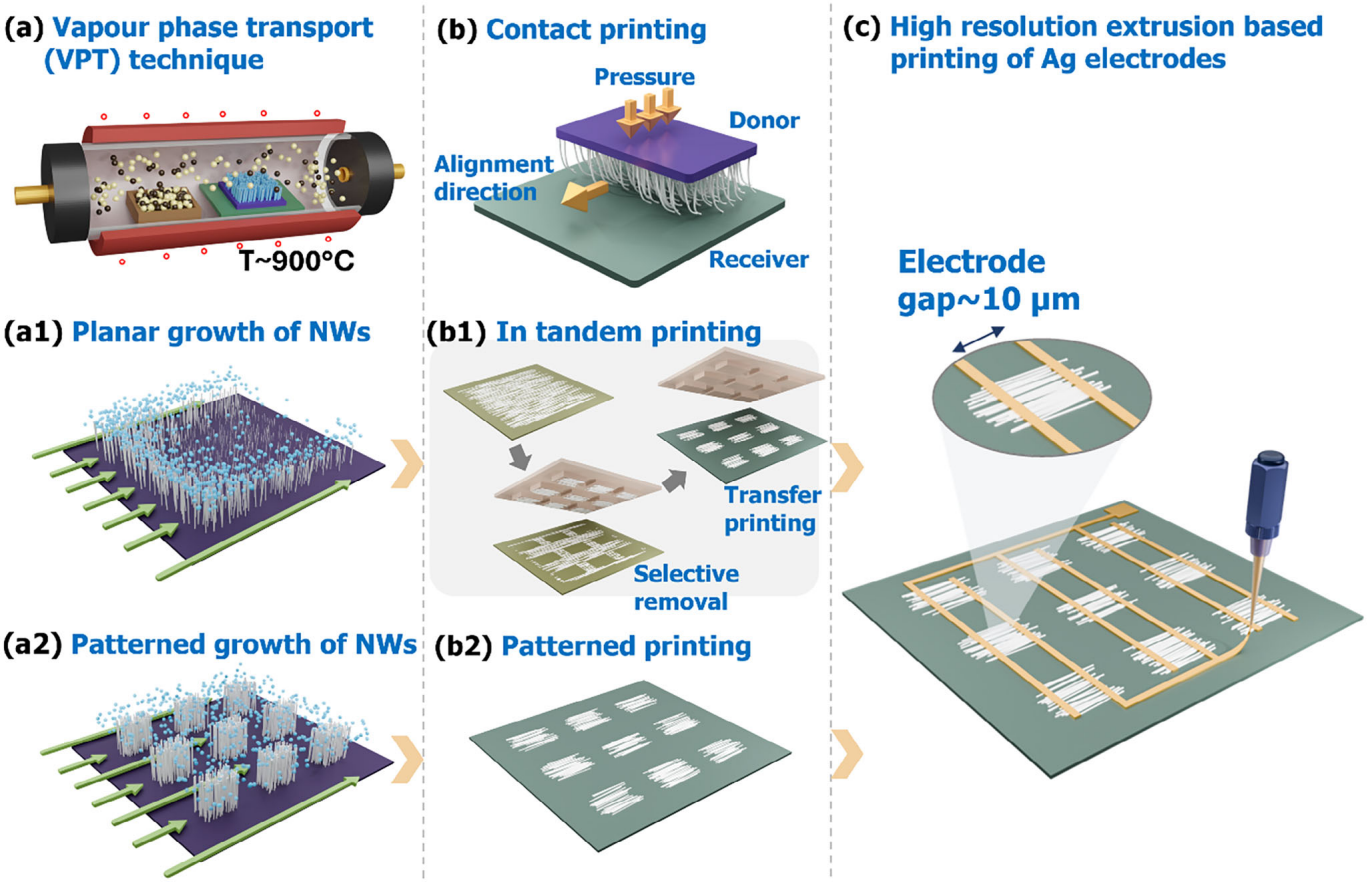
Schematic illustration of the process flow for fabrication of contact‐printed nanowire (NW) based devices. a) NW growth by vapor phase transport (VPT) technique, b) Contact printing method, c) Printing of Ag metal lines using high‐resolution extrusion printer. a1) Growth of NWs in a planar configuration. b1) Printing of continuous NW layer and subsequent patterning steps using a combination of transfer and contact printing methods. a2) Patterned growth of NWs. b2) Contact printing of NWs at selected locations directly from the NW patterns grown as selected locations.

Printed electronics could potentially provide such a solution. Taking advantage of the van der Waals interaction, the synthesized NWs could be relocated from the donor to the target substrate, thus decoupling the high temperature synthesis from the thermally sensitive flexible substrates.^[^ ^16^, ^17^, ^18^, ^19^, ^20^ ^]^ For example, we have shown the in‐tandem contact/transfer printing as a viable strategy developed devices as selected location while reducing the overall dependence on lithography.^[^ ^14^, ^21^ ^]^ However, most of these methodologies cannot completely eliminate the lithography steps, as it is needed either for patterning the transfer printed NWs or for creating the metal contacts. Addressing this challenge, herein, we propose a “design to fab” strategy in which the device/circuit layout is implemented at the NW synthesis level itself (**Figure** **1**). The scheme in Figure 1 illustrates a three‐step process: (a) NW growth, (b) contact printing, and (c) device fabrication using printed metal lines. In the case of conventional growth, the NWs are synthesized all over the surface (Figure 1(a1)). Due to this, the NWs are printed all over the receiver substrate during contact printing (Figure 1(b1)). To obtain patterned NW arrays at selected locations using this route, additional processing is needed at b1 stage which usually requires lithography and etching steps. An example of such processing is the in‐tandem contact printing method (b1) where a polydimethylsiloxane (PDMS) transfer stamp is employed to pick the ensemble of NWs and transfer them to the defined locations on the receiver substrate. On other hand, the patterned growth of NWs (Figure 1(a2)) allow direct contact printing of NWs at defined locations only (Figure 1(b2)), thus simplifying the device fabrication. After having ensemble of NWs at defined locations, the electrodes are realized using a high‐resolution printing of silver inks on top of NWs (Figure 1c). This is in sharp contrast with conventional process flow which starts with NW synthesis, followed by device design, patterning, and further processing (e.g., metallization) to fabricate the device. The NWs are typically synthesized using methods such as hydrothermal, vapor‐liquid‐solid (VLS) growth etc.^[^ ^22^ ^]^ For optimal transfer of NWs patterns using contact printing approach, the desired length of NWs is in 25–50 µm range, for which VLS growth is most suited. VLS growth method also offers better dimensional control and site specificity than other methods. This eliminates the need for lithography and etching, thus reducing the environmental impact arising from the manufacturing process. The presented approach also reduces considerably the number of process steps with respect to conventional methods (Figure 1). As a proof of concept, we synthesized 5 × 5 array pattern and micro‐pad array patterns of ZnO NWs on Si substrate using VLS method and dewetted Au catalyst (≈100 nm diameter) at 900 °C. These two patterns were subsequently contact printed on flexible polyamide substrates to develop UV sensors which could detect UV light having intensity in the range of 10–50 mW/cm^2^. We observe that compared to the conventional non‐patterned growth and blanket NW printing, the presented work shows a notable improvement in the spatial uniformity over several critical NW metrics including NW length, diameter, density, etc.

## 2 Results and Discussion

Site‐specific semiconducting NWs with controlled dimensions were grown here by bottom‐up approach at temperatures >900 °C. The Vapor–Liquid–Solid (VLS) method was used here for synthesis of ZnO NWs on Si substrate by patterning Au catalyst pads with the gap between them varied from 40–2000 µm, leading to an increased uniformity of growth in comparison with planar growth (**Figure** **1**(a1).^[^ ^10^, ^23^ ^]^ The vertically grown NWs are commonly printed on flexible substrate using contact^[^ ^17^, ^24^, ^25^, ^26^ ^]^ or transfer printing^[^ ^25^, ^27^, ^28^ ^]^ to obtain electronic layers with horizontally aligned NWs, as shown in Figure 1(b2)  for comparison. In our previous work, these layers are selectively removed using polymer stamps to develop devices as per the design and layout (Figure 1(b1).^[^ ^29^, ^30^, ^31^, ^32^ ^]^ By contrast, the current work is designed to obtain certain patterns of NWs at selected locations during the growth process itself by pre‐patterning the catalysts on the substrate (Figure 1(a2). In doing so, we reduced several fabrication steps. The materials usage is lowered, and implementation of device design starts at the material growth stage itself. The location selectivity is achieved due to the patterned presence of catalyst particles and the areal uniformity of growth manifested with the ability of vapor flux to access catalyst particles efficiently. The NWs were then contact printed on rigid and flexible substrates and the printed array of NWs was further processed to fabricate photodetectors for UV sensing.

### 2.1 Nanowires Synthesis

**Figure 2:**
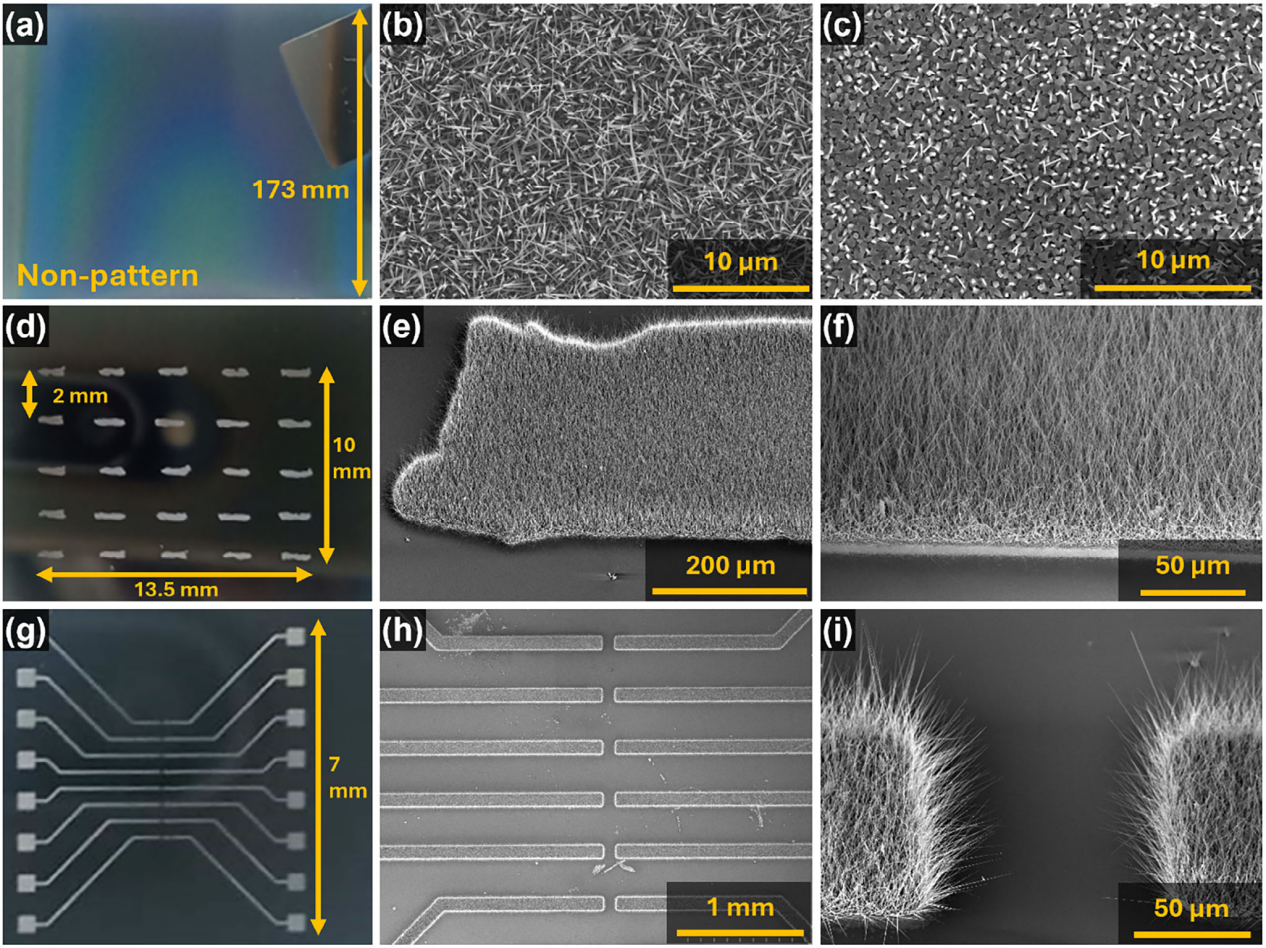
Photographs a,d,g) and SEM images comparing the patterned growth of ZnO NWs with planar continuous catalysts under similar conditions a–c) Planar growth showing NWs b) and c) mixed morphologies. d) Photograph showing all 25 sites where NWs were grown. e,f) SEM images of section of pattern show dense growth of NWs. g–i) micro‐pad shaped pattern of NWs. h) SEM image showing uniform growth of NWs over the patterns. i) SEM image depicting growth within a gap of ≈100 µm.

**Figure 3:**
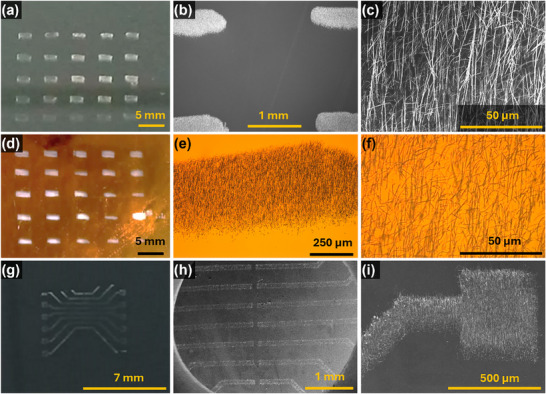
Photos a,d,g) and SEM images of printed NWs patterns on Si a–c,g–i) and Polyamide (PI) substrates d–f. a) photo of 5 × 5 array of NWs printed on Si, b,c) SEM images of printed array. d) 5 × 5 printed array on PI, e,f) optical micrographs of the printed NWs on PI. g–i) photo and SEM images of the printed micro‐pad pattern on Si.

ZnO NWs were grown using Au particles as catalyst using vapor phase transport (VPT) technique under inert Ar carrier gas ambient in a VLS approach.^[^ ^33^, ^34^, ^35^ ^]^ The NWs were grown three different configurations (**Figure** **2**): 1) NWs grown everywhere (i.e., no pattern) of planar Si surface with Au catalyst particles present all‐over (Figure 2a–c), 2) NWs grown at sites forming a 5 × 5 array pattern in an area of 135 mm^2^ and 2 mm spacing between the growth sites (Figure 2d–f), 3) NWs grown as parallel micro‐pad pattern with interspacing in the range of 100–300 µm (Figure 2g–i). The VLS process offers site selective growth of NWs by pre‐placing the catalyst particles on the substrate prior to growth of NWs. The 75–150 nm diameter Au catalyst particles used in this work were prepared by dewetting process on Si substrate at temperature of 900 °C. These substrates were positioned downstream of the vapor source, where Zn and O species were generated by heating a mixture of ZnO powder and graphite. The careful placement of Au nanoparticles decides the location of NW growth and the size of catalyst particle determines the diameter of NWs. The elemental Zn atoms from vapors are selectively injected through the Au catalyst particles and converted into NWs by crystallizing at the catalyst/substrate interface using the oxygen in the growth ambient. The growth was carried out at a temperature of 900–950 °C for 60–120 min, with a flow rate of Ar carrier gas of 750 sccm. Zn vapors created by the carbothermal reduction mechanism were transported to the vicinity of the solid Au surface using the Ar carrier gas and the continuous incorporation of elemental Zn into solid Au catalyst particle aided the creation of AuZn liquid droplet that led to supersaturation, which was followed by the crystallization of ZnO NWs at the interface. This led to steady growth of NWs in the vertical direction and continued so long as the Zn vapors were generated and ambient oxygen was available. In the case of (non‐patterned) planar NWs growth process, the Au catalyst particles with typical interspacing of 10–50 nm were formed (due to Au thin film dewetting process) over large area for NWs growth. Under VPT conditions, ZnO NWs generally did not grow uniformly, as shown in the photograph (Figure 2a), despite the presence of Au catalyst particles. The non‐uniformity is apparent from the white areas shown in the image. The NWs density in such areas is high (Figure 2b). The violet with graded colored regions in Figure 2c indicates a mixture of large ZnO crystals with partial growth of NWs. In this experimental condition, a variety of ZnO morphologies were observed at different locations of the substrate (Figure S1, Supporting Information).

Uniform areal growth of NWs is a prerequisite for their efficient printing (details in the next section) and subsequent device fabrication. It is important to eliminate the barriers leading to non‐uniform growth of NWs so as to attain high growth yield (NWs grown/number of catalyst particles).^[^ ^36^ ^]^ In the initial phase of NWs growth process, the catalyst particles, exposed to Zn/O vapor, undergo a transition from solid to liquid to overcome an energy barrier.^[^ ^37^, ^38^, ^39^ ^]^ Once the catalyst particles convert to nano‐liquid solution, the adsorption barrier for Zn atoms decreases due to rough (catalyst) liquid surface to efficiently accommodate the atoms from vapor. However, owing to insufficient saturated vapors at various catalyst locations, there is no uniformity over the catalyst particles during this initial phase. The constant flow of vapor precursors over the catalyst particles, due to pressure difference between inlet and outlet of reactor tube, also does not allow sufficient time for the vapors to stay in the vicinity of the catalyst particles. The high temperature conditions also favor the growth of ZnO crystals directly over the NWs surface, which affects the 1D growth process (although lateral crystal growth is enhanced). These factors result in large ZnO crystals at the base of the NWs and also the lateral growth in the areas between the catalyst particles. An efficient strategy is needed to overcome these issues to obtain uniform NWs growth all over the catalyst particles.^[^ ^40^, ^41^, ^42^, ^43^, ^44^ ^]^ Under the similar planar growth conditions (similar substrate size and growth parameters), the areal reduction of catalyst particles leads to increasing vapor‐catalyst interaction.^[^ ^45^ ^]^ In this context, a 5 × 5 array of rectangular shaped (area≈0.6 mm^2^) catalyst particle patterns were used over an area 135 mm^2^. Thus, 25 such patterns covered ≈11% of the substrate with uniformly grown NW (**Figure** **3**d). The vapor sources reaching all catalyst locations, during all growth stages, lead to uniform growth and distribution of NWs – thanks to the gaps between the patterns. The increment of the ratio of vapor source to number catalyst particles also contributes toward uniform growth of NWs. For example, this ratio is ≈0.3 when the catalyst particles are present all over the area of 135 mm^2^ and it increases to ≈0.8 in the case of 5 × 5 array even if the catalyst is only 11%. In the latter case, the higher amount of edges offer the critical entry points for vapors to play significant role in getting uniform growth of NWs. During early stages of non‐patterned NW growth, the edges could swiftly establish steady state growth and absorb more incoming flux, leading to source vapor scarcity for other regions. As a result of NWs growing at higher rate near the edges, the vapor sources are lower in other growth regions. Given this background, the 5 × 5 array pattern offers more uniform growth as there are more edges and hence more open pathways for the vapor flux to reach the catalyst at all stages of NWs growth. This has helped to attain ≈35 µm long NWs in the growth duration of 2 h (Figure 2e,f). The benefits of pattern enhanced uniform NWs growth is also validated with a micro‐pad electrode pattern (Figure 2g). In this pattern, the gap between electrode varies in 100–2000 µm range and uniform growth can be noted (Figure 2h,i) over all segments with the same growth conditions. This verifies that the presence of edges is a key factor leading to uniform growth over a defined area. Thus, the patterned growth of NWs offers a better alternative for obtaining electronic/sensing layers, as besides offering a lithographic‐free process, it also offers better uniformity among NWs growth areas and hence better conditions for large area contact printing technique, as described in the next section.

### 2.2 Printing of Nanowires Based Electronic Layer

The 5 × 5 array and micro‐pad pattern of NWs were printed on Si (Figure 3a–c) and polyamide substrates (Figure 3d–f) using our custom contact printing method. Details of automated contact printing set up are available elsewhere.^[^ ^46^, ^47^ ^]^ Briefly, the contact printing process involves following sequential steps, a) the substrate on which NWs are grown (i.e., donor substrate) is brought in contact with a receiver substrate and an optimal normal pressure is applied uniformly between the substrates; b) The receiver substrates slide horizontally at an optimal speed. As a result of sliding, the free end of NWs experiences tangential force which leads to their breakage from the donor substrates and printing along the direction of sliding. The NWs on the receiver substrate are quasi‐aligned along the direction of sliding. The contact printing set up used here allows self‐alignment of donor‐receiver substrates to ensure conformal contact between them. This is critical for the uniform transfer of NWs from donor to receiver substrate. In this work, this conformal contact is over an area of ≈645 mm^2^(total size of donor sample) (Video S1, Supporting Information). The overall dimensions of printed NW patterns are ≈135 mm^2^ (for 5 × 5 rectangular array (Figure 3a,d) and ≈50 mm^2^(for micro‐pad pattern (Figure 3g)). The spacing between various regions of printed patterns varies between 100–2000 µm. For the case of 5 × 5 array, the donor‐receiver pressure during sliding was 240 kPa, the sliding speed was 100 µm s^−1^ and the stroke was 500 µm. The above pressure was generated by the force of 4.5 N applied on the donor stage for the total sliding period of ≈16 s (Supporting Figure S2; Video S1, Supporting Information). The printing parameters of non‐patterned NWs (i.e., NWs presented all over the growth substrate) in an area of ≈100 mm^2^ was 33 kPa, the sliding speed was ≈100 µm s^−1^ and the stroke was 5 mm. In comparison to non‐patterned NWs, the pattered NWs required 8 times higher pressure to ensure uniform printing of NWs. The transfer of the as grown NWs pattern, shown in Figure 3a,d,g, demonstrates the location specific printing without employing the conventional lithography steps such as pre‐patterning and chemical functionalization of the receiver substrate.^[^ ^17^, ^48^ ^]^ By initiating the implementation of the design/layout of devices at pressure to ensure uniform printing of NWs. The transfer of the as grown NWs pattern, shown the NW synthesis level itself, the presented method eliminates several fabrication steps that typically require chemicals such as photoresists, developers etc.

In the 5 × 5 array of NWs, only 11% of the 135 mm^2^ area is covered with NWs (≈30‐35 µm long) as against 100% area in the case of non‐patterned growth of NWs. This means that ≈ 85% of the donor wafer is not in contact with the receiver (gap between the two substrates is equivalent to the length of NWs, ≈30 µm) during the beginning of printing process. This gap increases to ≈100 nm (i.e., diameter of NWs) soon after the printing or sliding process starts. Thus, there is an opportunity to tune the interfacial pressure and enhance the printing yield simply by engineering the dimensions of growth pattern. It may be noted that the sliding motion also causes a horizontal shift of the patterns in the alignment direction and this is equivalent to the length of NWs. This is apparent in the photographs shown in Figure 3d,i as elongations in the sliding direction. This information is critical for subsequent steps such as printing or deposition of conductive layer to build devices based on NWs‐based printed electronic layers.

**Figure 4:**
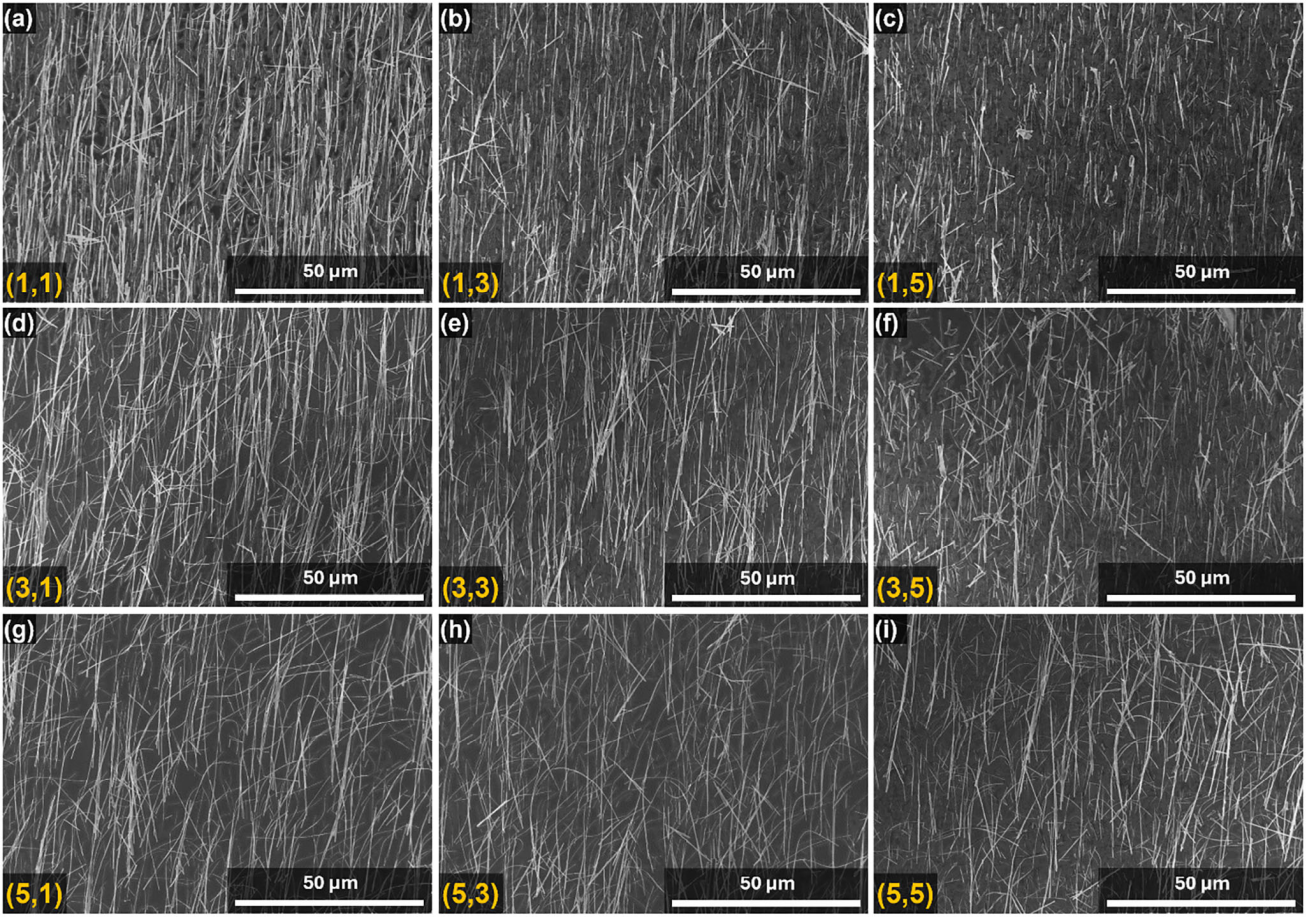
SEM images of contact printed ZnO NWs as 5 × 5 array pattern with schematic replica of the receiver substrate (middle). a–c) SEM images of row‐1‐, d,e) row 3, f–h) row 5. All the images contain same scale bar of 50 µm for comparison.

The patterned printing could also prevent the piling of NWs, particularly if the length of NWs is lesser than the gaps between NWs growth sites. This has been a major issue when non‐patterned NWs are printed onto receiver substrate. In the case of 5 × 5 patterned NWs array, the pattern width of ≈300 µm and sliding stroke of 100 µm s^−1^ helps to accomplish the printing within 5 s with adequate adhesion of NWs over receiver substrate (Video S1, Supporting Information). The key figure of merits of the printed NWs are monolayer printing, length uniformity, oriented alignment, and constant density over an area. The statistical analysis 5 × 5 array of NWs pattern was carried out using SEM images of all the pads. **Figure** **4** shows the SEMs images of rows 1,3 and 5 of the 5 × 5 array pattern at same magnification and scale bar. The monolayer assembly, density, alignment and orientation of the NWs across all the patterns largely resemble. For the subsequent development of printed devices, length of NWs and alignment across the area are crucial figure of merits. The precision of the 5 × 5 array and micro‐pad patterns of printed NWs demonstrated in this work lies within the range of 100–2000 µm. However, the interspacing between patterns can be lowered to below 100 µm range by redesigning the growth patterns, taking into account the NW length‐to‐pattern interspacing factor in the design criteria.

**Figure 5:**
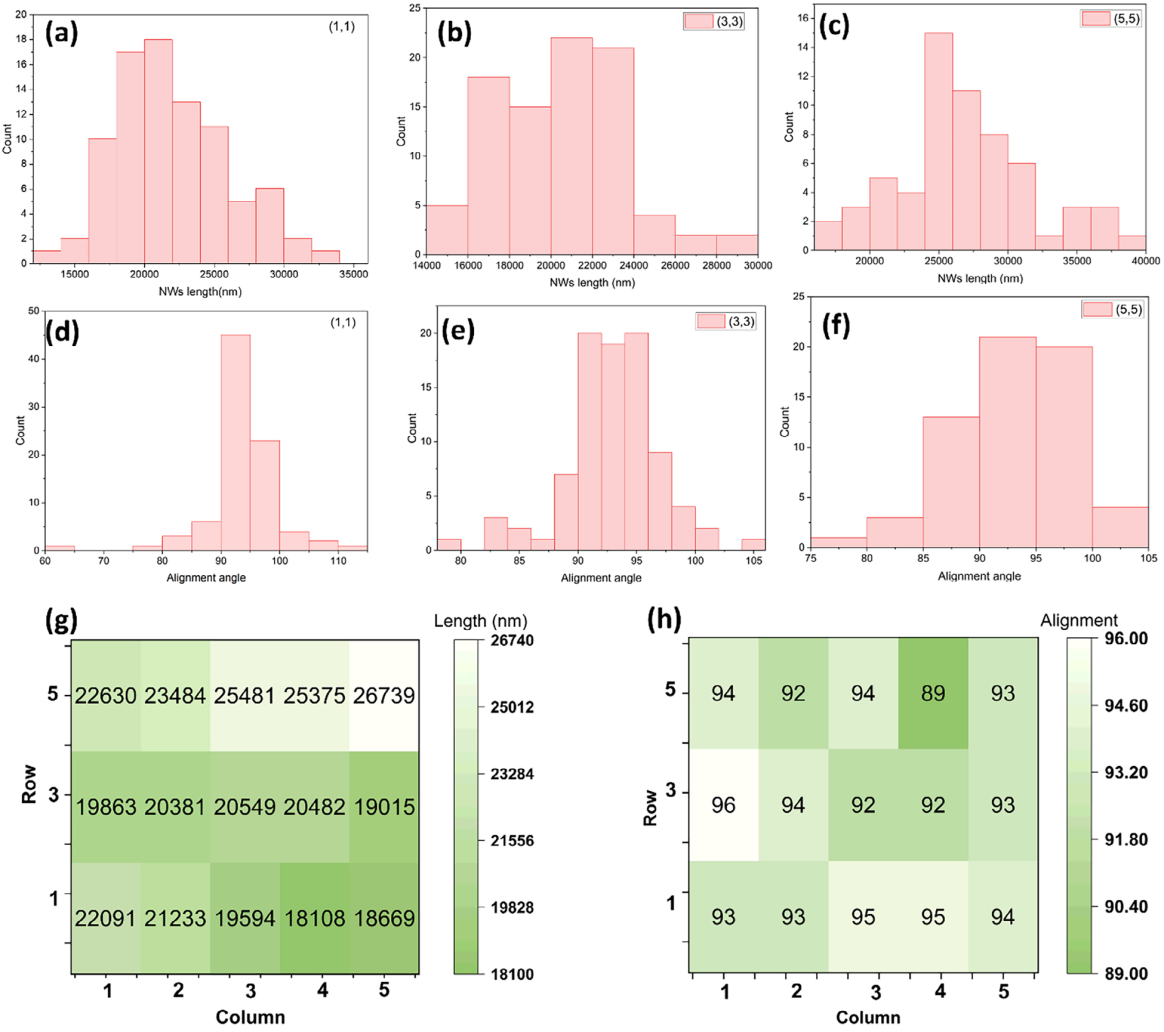
Statistical analysis of length distribution and alignment of the printed patterns (rows 1,3 &5). a–c) histograms of length distribution of patterns (a)‐(1,1), (b)‐(3,3), (c)‐(5,5). d–f) histograms of alignment angle of the patterns (d)‐(1,1), (e)‐(3,3), (f)‐(5,5). g) map of average length of NWs across rows 1,3 &5. h) average orientation angle of all the patterns in rows 1,3 &5.

Multiple SEM images of each pattern were analyzed (≈200 images across all sites) to quantify length and alignment of the printed NWs (**Figure** **5**). The statistical analysis of rows 1, 3 and 5 shows the average length of NWs and the alignment angle vary between 18–26 µm and 88–96° respectively. The histograms of NWs length distribution across all the sites of the array depicts high printing yield (i.e., 100% pattern transfer) with uniform alignment. This could be attributed to the conformal contact between donor and receiver substrate and the quality of NWs. The detailed histograms of each NW pattern site is given in Figs. S3.1 and S 3.2. The length distribution across the cell shows a ≈50–75% retention of the original length NWs after the printing process. This is sufficient for devices such as FETs, sensors etc., which typically require active channel length in the order of 1–10 µm.

### 2.3 Fabrication and Characterization of Printed UV Sensor

**Figure 6:**
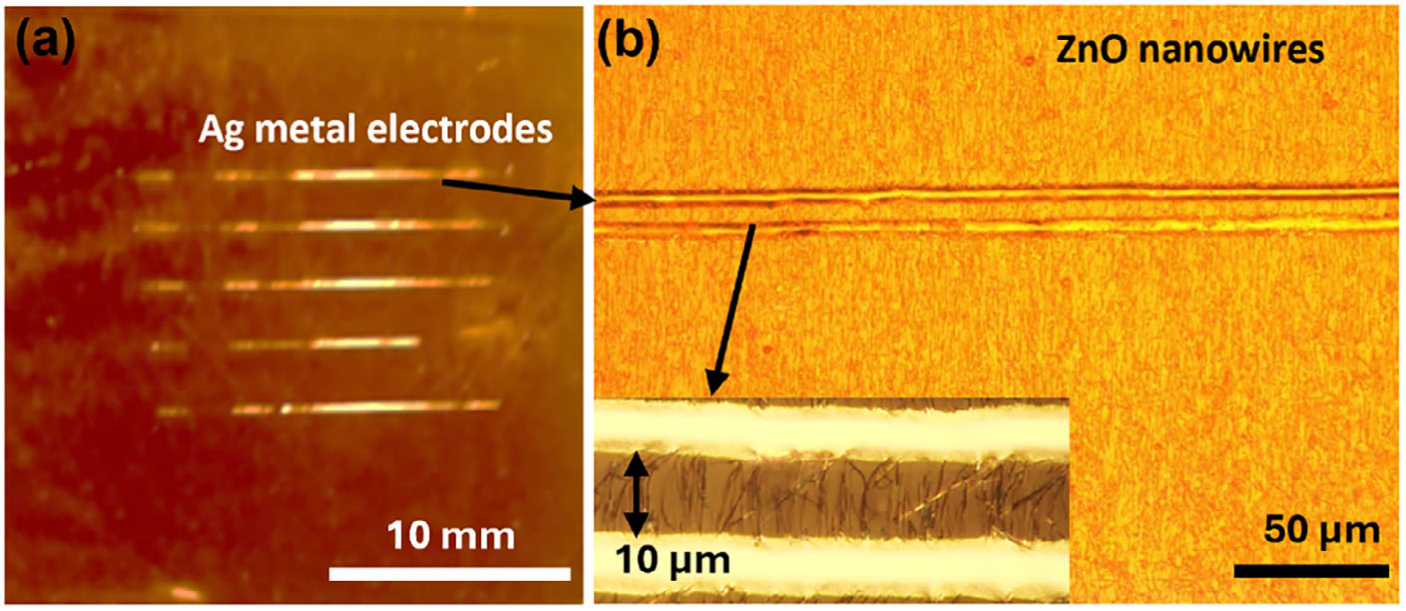
Images of silver (Ag) metal electrodes printed over a 5 × 5 array of ZnO nanowires (NWs) to develop the flexible UV sensor on polyimide (PI) substrate. a) Photograph shows printed silver electrodes connecting NWs in all five patterns in each row. b) Higher‐magnification optical micrograph showing on location of ZnO NWs with 5 µm wide Ag electrode printed at a gap of ≈10 µm.

**Figure 7:**
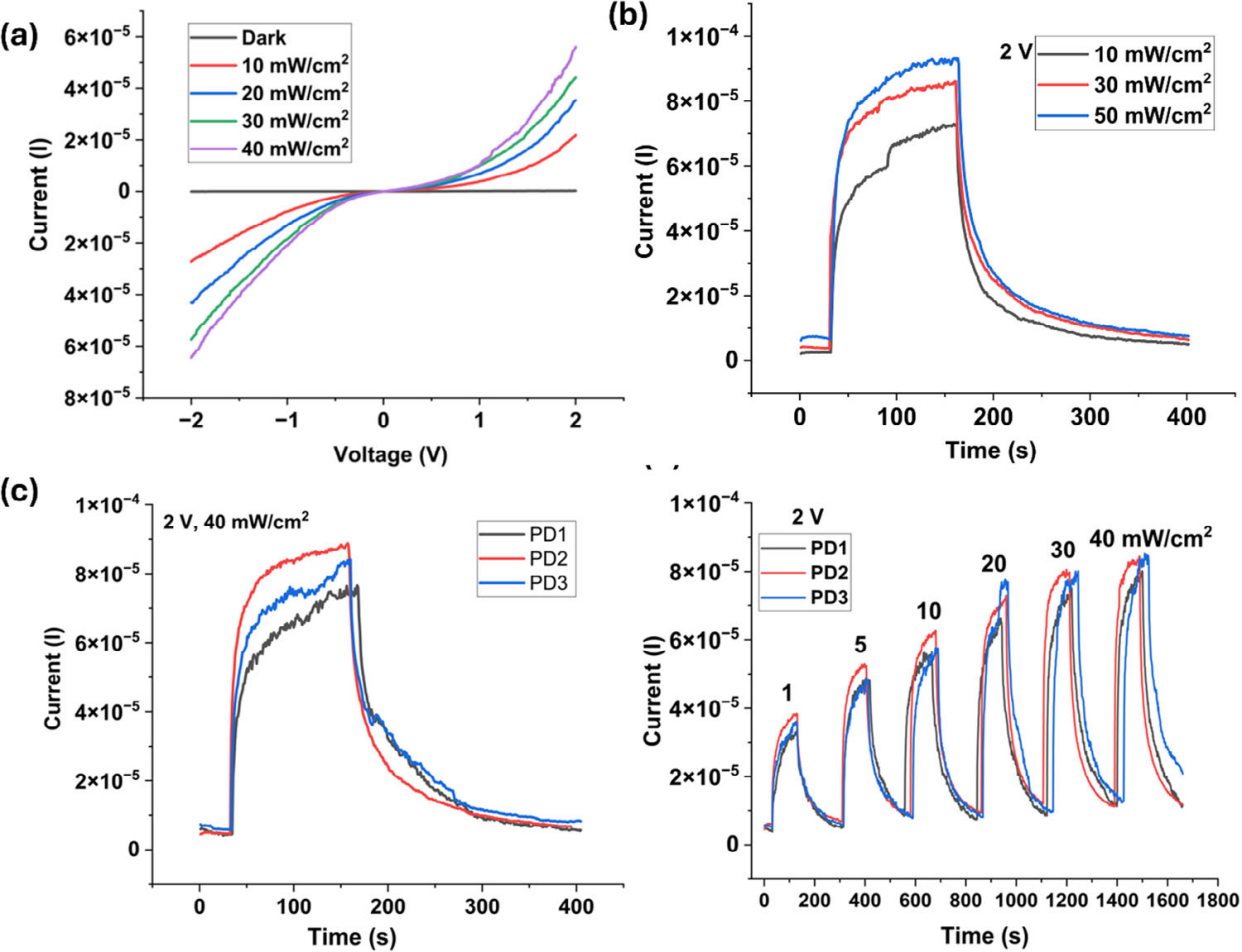
Electrical characteristics of printed photodetectors. a) *I–V* characteristics under the dark and various illumination intensities in the voltage sweep between ±2. b)Transient photo response of the PDs at different UV intensities in the range of 10–50 mW cm^−2^ at 2V. c) Transient characteristics of 3 different PDs at 2 V and 40 mW cm^−2^. d) temporal response of 3 PDs at 2 V in the range UV illumination 1–40 mW cm^−2^.

We fabricated large‐area, flexible UV photodetectors (PDs) based on printed patterned arrays of ZnO nanowires (NWs) (**Figure** **6**). High‐resolution silver (Ag) metal electrodes were printed onto the ZnO NW array on a flexible PI substrate using an extrusion‐based printing system (XTPL Delta). We performed a detailed electrical characterization of the PDs fabricated by patterned contact printing, as shown in **Figure** **7**. Figure 7a presents the current‐voltage (I‐V) characteristics of the PD under various UV illumination intensities, ranging from 0 (dark) to 50 mA cm^−2^ at a wavelength of 365 nm. Upon illumination, the ZnO NWs absorb UV light and generate photocarriers and the resulting photocurrent increases with increasing illumination intensity due to the enhanced generation of photocarriers. The symmetrical, non‐linear I‐V response can be attributed to the formation of Schottky contacts between the n‐type ZnO NWs and the Ag metal electrodes.^[^ ^49^ ^]^ The generated photocarriers are separated by the applied external electric field and collected at the printed electrodes, leading to a measurable photocurrent. The high surface‐to‐volume ratio of ZnO NWs results in many surface states that can act as hole traps. This leads to a wide range of carrier relaxation dynamics, spanning in the timescale of 10^−9^ to 10^2^ s. Under UV illumination, the high density of photogenerated holes discharge the negatively charged adsorbed oxygen molecules on the surface and subsequently the photogenerated electrons are collected at the electrodes. The enhanced surface area facilitates hole trapping and oxygen adsorption‐desorption kinetics, significantly contributing to the high photocurrents and sensitivity observed in ZnO NW photodetectors.^[^ ^49^ ^]^

The PDs exhibited a dark current of 1.5 µA and photocurrents of up to 80 µA under a bias voltage range of −2 V to +2V. Figure 7b illustrates the transient photo response of the PD at a 2 V bias voltage under varying UV light illumination. A device has a dark current of 1.5 µA and a photocurrent of 76 µA under 40 mW cm^−^^2^ illumination at a 2 V bias, resulting in an ON/OFF current ratio of 52. The device was exposed to UV light intensities ranging from 10 to 50 mA cm^−^^2^ for ≈120 s to reach current saturation, followed by a recovery period. The device exhibited response and recovery times of 25 and 60 s, respectively, at 2 V and a UV intensity of 40 mA cm^−^^2^. These relatively long response and recovery times can be attributed to the large active area of the printed PDs and high UV intensity that causes persistent photoconductivity in ZnO NWs.^[^ ^50^, ^51^ ^]^

The temporal photo response of the devices was measured under UV illumination intensities ranging from 1 to 40 mW cm^−^^2^ at a biasing voltage of 2 V, as shown in Figure 7d. The PDs exhibited incremental photocurrent responses with increasing UV illumination, with peak currents ranging from 4 to 85 µA. To evaluate the impact of printing uniformity on device performance, three devices from rows 1 to 3 were tested for transient photo response at 2 V and 40 mA cm^−^^2^ (Figure 7c,d). The results indicate consistent temporal performance across multiple devices, with peak current variations within an order of magnitude at each designated UV illumination level (Figure 7d).

**Figure 8:**
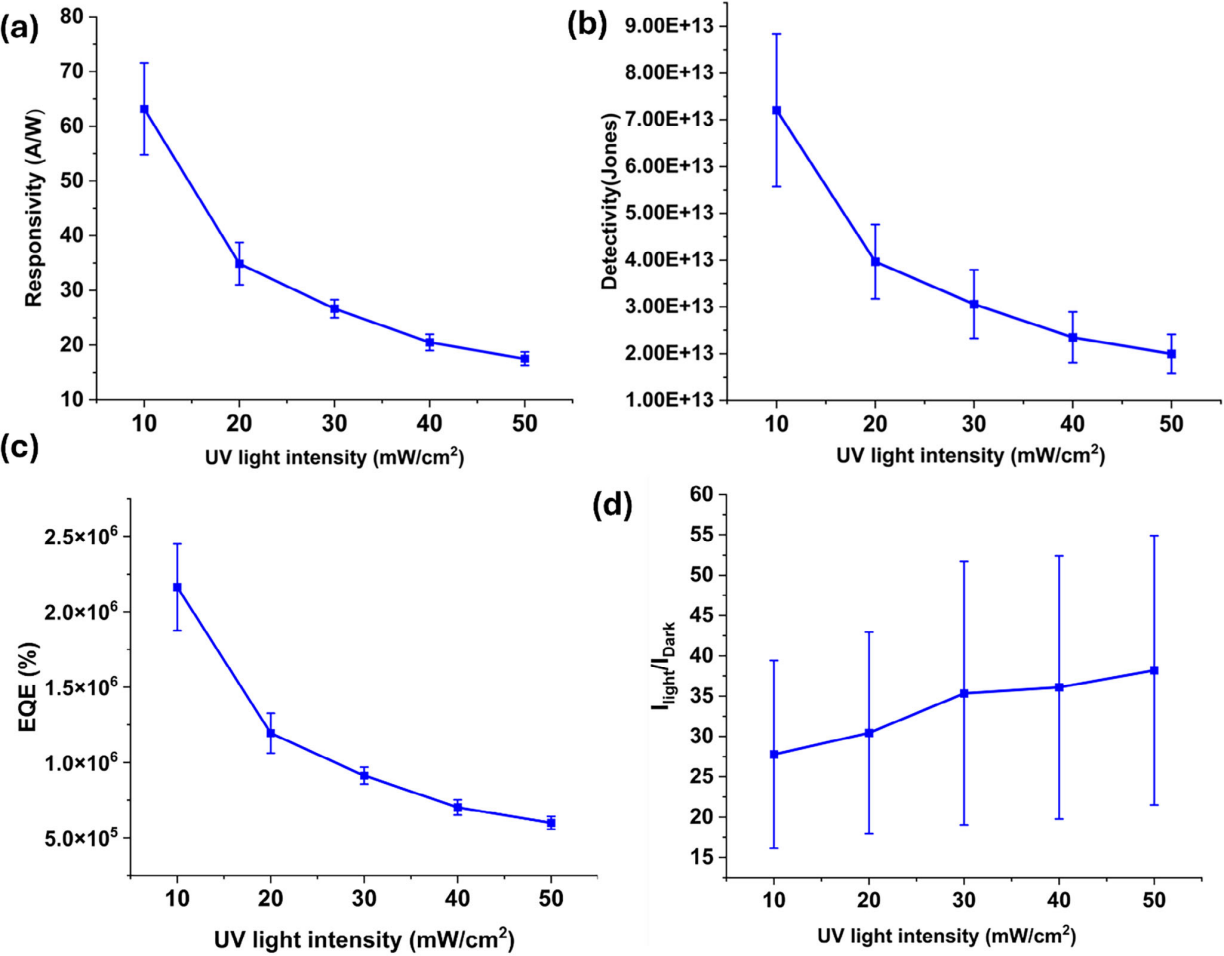
Evaluation of Figure of merits of printed PDs and statistical analysis of a) responsivity, b) detectivity, c) external quantum efficiency (EQE) d) ON/OFF ratio

To quantify the performance of the PD further, the important parameters such as responsivity (*R*), detectivity (D*), external quantum efficiency (EQE), and ON‐OFF current ratio (I_Photo_/I_Dark_) were extracted from the time‐resolved photo response at varying illumination intensities. The statistical data of these figure of merits of three PDs are presented in **Figure** **8**.

The *R* of a PD is defined as the ratio of the photogenerated current to the illumination power intensity:^[^ ^52^ ^]^

(1) $$R=\frac{\left(I_{Photo}-I_{Dark}\right)}{P_{in}\;\times \;S}\;$$

where *I_Photo_* is the photogenerated current, *I_Dark_* is the current under dark conditions, *P_in_* is the UV illumination power per unit area, and *S* is the effective PD sensing area. Figure 8a shows the *R* under various exposed intensities between 10–50 mW cm^−2^, with a maximum of 72 A W^−1^ at 2 V biasing under 10 mW cm^−2^ of UV light intensity. The responsivity of all measured devices exhibited a monotonic decrease with increasing UV light illumination intensity. The lowest measured R was 17.5 A/W at 10 mW cm^−2^ with a standard deviation of 1.2. As shown in Figure 8a, the printed devices from different locations exhibited consistent photo response and uniform dependency across the range of UV light intensities. Further, detectivity, D* is defined as:

(2) $$D^{*}=\frac{R}{\sqrt{2qJ_{Dark}}}$$

where *q* is the charge of the electron and *J_Dark_* is the dark current density. D* provides a quantifiable measure of the smallest detectable optical signal that the device can perceive and is commonly regarded as a crucial figure of merit for photodetectors. The PD displays a maximum detectivity of 7.2 × 10¹^3^ Jones with a standard deviation of 1.6 × 10¹^3^ at a UV light intensity of 10 mW cm^−^^2^. The high detectivity can be attributed to efficient light trapping by the large surface area of ZnO NWs, which multiplies the photocurrent through various mechanisms, including an extended light path along the length of NWs and multiple internal reflections within the NW structure.^[^ ^53^ ^]^ The high aspect ratio of NWs increases the optical path length within the semiconductor and leads to improved optical absorption and consequently, an increase in photocarrier generation. The enhanced light trapping in photodetectors (PDs) could also be attributed to the increased optical absorbance due to Schottky contacts formed with Au electrodes. Compared to ZnO thin films, the improved light trapping within NWs significantly enhances the detectivity of NW PDs.^[^ ^54^ ^]^ Like the responsivity, the detectivity also exhibits a decreasing trend with increasing UV intensity, reaching a minimum value of 1.9 × 10¹^3^, as shown in Figure 8b. The EQE of the PD, which is a measure of how much‐absorbed photons were converted into useful photocarriers, is given as^3^:

(3) $$EQE=\frac{hc\;\times \;R}{e\lambda }\;\;\times 100$$

where *h* is Planck's constant, *c* is the velocity of light, and λ is the wavelength of incident light. The PD has a maximum EQE of 2.3 × 10^4^% for the UV light intensity of 10 mW cm^−2^ with a standard deviation of 2.7 × 10^3^% (Figure 8c). The higher EQE of ZnO NWs could be attributed to the large internal gain (≈10^8^), which arises from various photomultiplication mechanisms^[^ ^59^ ^]^ including impact ionization, oxygen adsorption‐desorption kinetics at the surface, localized electrical field enhancements, and photon trap‐assisted processes. Additionally, the *I_photo_/I_dark_* ratio, extracted from the transient photoresponse measurements, ranged from 27 to 38 under illumination intensities of 10 to 50 mW cm^−^^2^ at a 2 V bias. The low *I_photo_/I_dark_* ratio is primarily attributed to the large sensing area (9.75 × 10^−5^ cm^2^) of the printed NWs patterns. The large sensing area is due to the use of large number of NWs. The enhanced surface area of the ZnO NWs introduces more charge carrier trapping states due to the presence of point defects, leading to a current flow even in the absence of UV light. The *I_photo_/I_dark_* ratio can be significantly improved by reducing the diameter of the grown nanowires and optimizing the sensing area through adjustments to the dimensions of the Ag metal electrodes within the printed nanowire pattern. The extracted figure of merit and performance parameters of the PD were compared to those of other ZnO‐based printed UV detectors and are summarized in **Table** **1**. The table presents various fabrication methodologies for ZnO NW PDs, including contact printing with additional patterning strategies and drop‐on‐demand metal electrode printing, as well as conventional solution‐based NW printing techniques. In comparison, this work demonstrates a two‐step lithography‐free dry fabrication process for producing large‐area PD arrays that achieved figures of merit at par with the devices fabricated using more complex methods. The PDs in this work utilized large‐area patterns to demonstrate the scalability of the patterned printing process. Each printed pattern resulted in a device with a significantly larger active area compared to previously reported devices.^[^ ^14^, ^21^ ^]^ The active device area and UV illumination intensity are crucial factors that influence the device's performance, including responsivity, *I_photo_/I_dark_* ratio, and response/recovery times. By varying the pattern dimensions, it is possible to tailor the device performance to specific application requirements. The presented method offers a promising approach for tailoring the performance of PDs through pattern‐dependent fabrication, potentially enabling resource‐efficient nanofabrication technique for sustainable electronics.

**Table 1:** Comparison of the PD performance with the state‐of‐the‐art printed PDs based on ZnO.

Materials	R [A/W]	D* [jones]	I_photo_/I_dark_	EQE [%]	Response/Recovery time [s]	UV [mW cm^−2^]	Remarks	Refs.
ZnO NWs	8 × 10^7^	8 × 10^16^	9	2.5 × 10^10^	15/30	1 × 10^−5^	In‐tandem contact transfer printing	[14]
ZnO NWs	1.9 × 10^5^	5.65 × 10^15^	885	6.1 × 10^7^	28/13	1 × 10^−5^	Contact printing	[21]
ZnO NWs	7.8 × 10^6^	1.67 × 10^16^	2410	2.6 × 10^9^	N/A	5 × 10^−5^	Contact printing	[55]
Graphene nanoplatelet/ZnO	2.2	2.03 × 10^11^	100 to 1000	N/A	9.6/17.2	2 × 10^−5^	Inkjet printing	[56]
ZnO NWs	7.5 × 10^6^	3.3 × 10^17^	>10^5^	N/A	0.56/0.32	5 × 10^−5^	Printing	[57]
ZnO/Ag NP	4.8 × 10^−3^	1.45 × 10^10^	N/A	N/A	3.9/3.3	0.28	Inkjet printing	[58]
ZnO NWs	5.8 × 10^3^	8.5 × 10^15^	38	2.3 × 10^4^	25/60	10	Patterned Contact printing	This work

## 3 Conclusion

The work demonstrated a NWs‐based fabrication process flow for high‐performance printed electronics. Patterned 5 × 5 array of ZnO NWs were grown using bottom‐up VLS mechanism through VPT technique over an area of 130 mm^2^ with individual rectangular cell dimension ≈0.75 mm^2^. The interspacing between the cells is 2 mm which enabled uniform growth of NWs over all the cells. NWs grown at ≈900 °C for 2 h produced length in the order of ≈35 µm over all the cells. The vertically grown NWs has been contact printed using an indigenously built automated system. The printed parameters have been analyzed in comparison with planar grown NWs of similar area that has brought out key aspects responsible for the patterned printing of NWs. The estimated printing pressure (240 kPa) is 8 times higher than non‐patterned planar NWs grown in the similar area (100 mm^2^) that has played as key enabler for high printing yield of 25 patterns. Further, multiple SEM imaging across 15 cells (rows 1,3 and 5) has been used to extract statistical data of the printed NWs patterns. The analysis helped to ensure the length uniformity range and alignment of the all the printed patterns. The printed patterns subsequently have been used to fabricate flexible UV photodetectors. Multiple PDs have been characterized under different illumination conditions and the figure of merits were extracted from the measurements. The performance of this fully printed sensor is in par with recently reported UV PDs fabricated using different printing approaches. As a fabrication methodology, the demonstrated process flow is fully dry and has not used expensive clean room‐based procedures. Importantly, the final device architecture can be decided in the materials growth/synthesis stage to gain more control in view of NWs based circuit development. Further, evolution of these techniques will help to reduce e‐waste and create degradable high‐performance electronic components in the future.

## 4 Experimental Section

### Patterned Growth and Characterization of ZnO NWs

ZnO NWs were grown by VLS mechanism using the vapor phase transport (VPT) method. Au catalyst particles of ≈100 nm diameter range were created by thin film dewetting process. Si substrates were cleaned using acetone, isopropyl alcohol (IPA) and deionized (DI) water prior to the deposition of patterned Au film. Stripes of Au film patterns (thickness≈5 nm) on Si deposited using metal and plastic hard masks through e‐beam evaporation. Similarly, for comparison of non‐patterned NWs growth, samples with continuous Au catalyst films deposited under similar conditions. The VPT growth system consists of a three‐zone furnace loaded with 3″ diameter quartz reactor connected to inert gases. A homogenous mixture of graphite and pure ZnO powder (Sigma Aldrich) kept at temperature on excess of 900 °C serve as source to create vapors for NWs growth under the flow of Argon career gas. NWs were grown using an equimolar weight of source powder (1:1 of graphite:ZnO) loaded on an alumina boat with Au catalyst/Si substrate placed in the vapor downstream. ZnO NWs grown using continuous and pattered catalysts have been characterized using Optical (Leica) and Scanning Electron Microscopy (SEM‐ Hitachi SU8240) techniques.

### Contact Printing of NWs

ZnO NWs are printed over rigid and flexible receiver substrates using an indigenously built automated contact printing (CP) system. The printing system consists of coupled vertical‐horizontal motorized stages which are precisely controlled using a NI LabVIEW interface. The Si substrate contains the grown NWs termed as “donor” mounted in the vertical stage facing the horizontal moving platform where the receive substrate was fixed. Donor‐receiver substrates were brought in contact followed by interface uniaxial sliding that led to the aligned printing of NWs. The interfacial exertion pressure (≈33 kPa) and sliding velocity (≈100 µm s^−1^) were the optimized crucial parameters to obtain an aligned high density of NWs.

### Fabrication and Characterization of Patterned Printed Photodetectors

The printed 5 × 5 array of ZnO NWs on PI foil has been used to develop a flexible UV photodetector (PD). The metal electrodes of the PDs were realized using direct ink writing (DIW) based high resolution extrusion printer (XTPL Delta printer) with gaps in the order of ≈10 µm which was effectively bridging the printed monolayer array of NWs. The highly viscous (>100000 cp) Ag silver ink (XTPL CL85) consist 82 wt% of metal nanoparticles (35–50 nm diameter) with shear rate of 0.2/s. To preserve the NWs alignment and structure, the printing nozzle has maintained gap of 1 µm above the monolayer while printing electrodes. The printing parameters such as extrusion pressure (7 bar), nozzle velocity (0.5 mm s^−1^), were optimized to yield smooth lines over NWs surface. The electrodes were annealed at 250 °C for 15 min to obtain good metal/semiconductor contact. A UV LED of 365 nm wavelength was used to as radiation source to study the photoresponse of ZnO NWs under different intensities. Electrical characteristics of the device was analysed using Agilent B1500A source meter.

## Conflict of Interest

The authors declare no conflict of interest.

## Supporting information



Supporting Information

Supplemental Video 1

Supplemental Video 2

## Data Availability

The data that support the findings of this study are available from the corresponding author upon reasonable request.
