# Supplementary material for: Site‐Specific Growth and Printing of Nanowires for Resource Efficient Fabrication of Flexible Electronics
Source: Small. 2025 Mar 17;21(17):2412685. doi: 10.1002/smll.202412685 (PMC12036555; doi:10.1002/smll.202412685)
Supplement: Supplementary file 1 — Supporting Information [file SMLL-21-2412685-s003.docx]

**Site-specific Growth and Printing of Nanowires for Resource Efficient Fabrication of Flexible Electronics**

D. Shakthivel, A. Christou, F. Liu and R. Dahiya

Bendable Electronics and Sustainable Technologies (BEST) Group, Electrical and Computer Engineering Department, Northeastern University, Boston, MA 02115, USA

S1. Planar NWs growth regions of various morphologies.


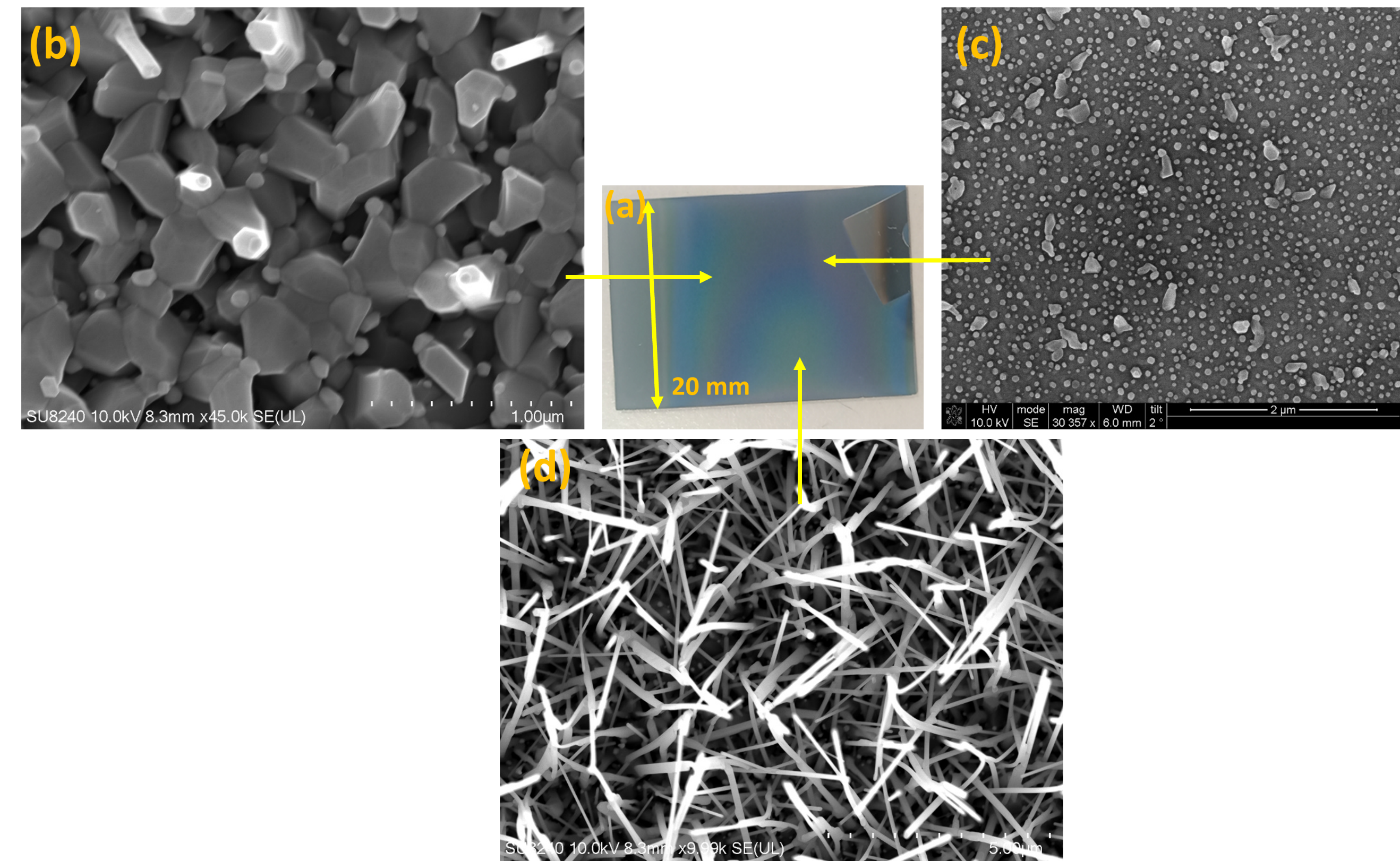


Fig.S1. SEM images of various regions of the ZnO NWs grown on 100 mm^2^ area

S2. Contact printing process parameters.


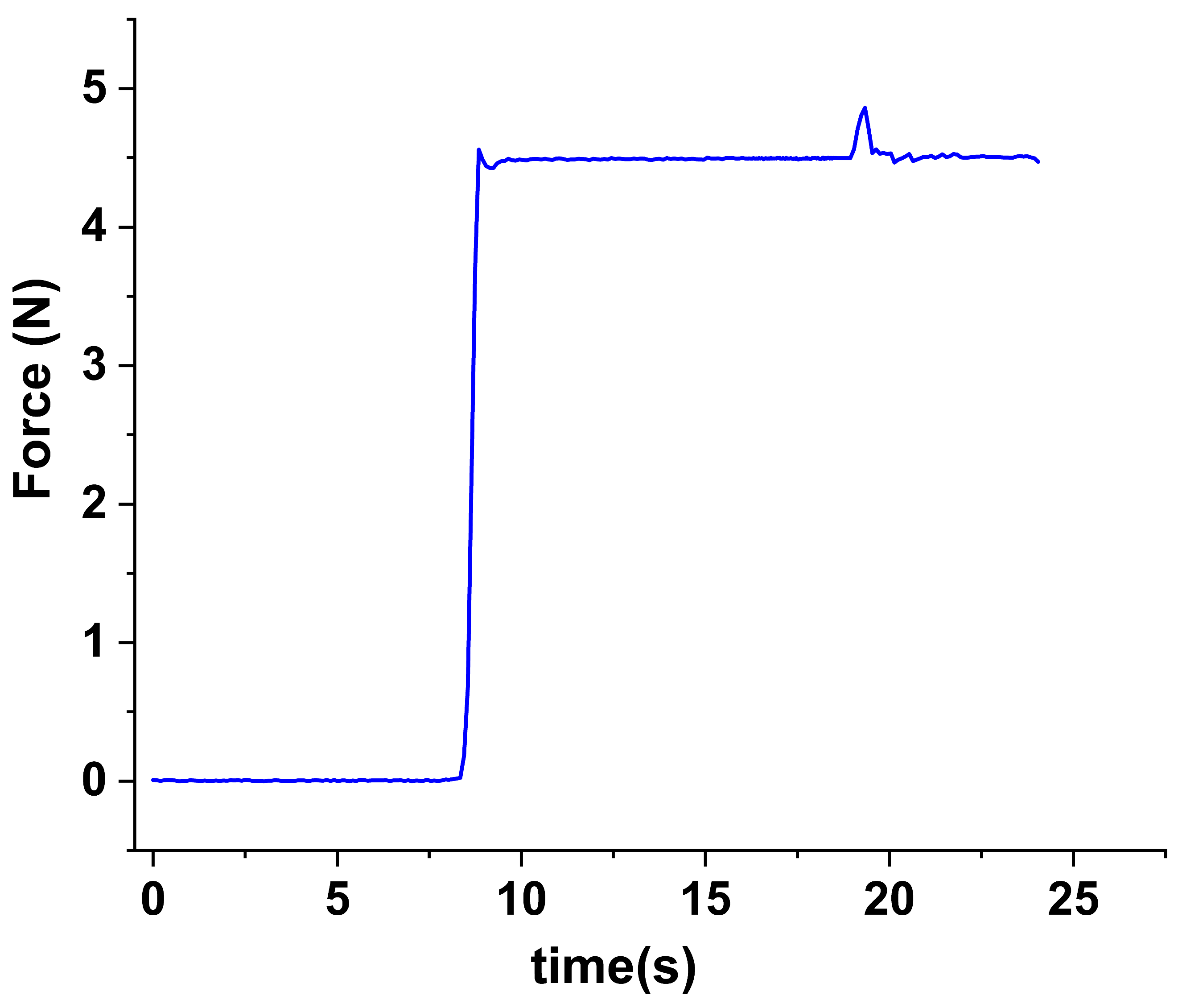


Fig.S2. Display of the applied force during the printing process

S3. Statistical analysis of the printed NWs of 5x5 array


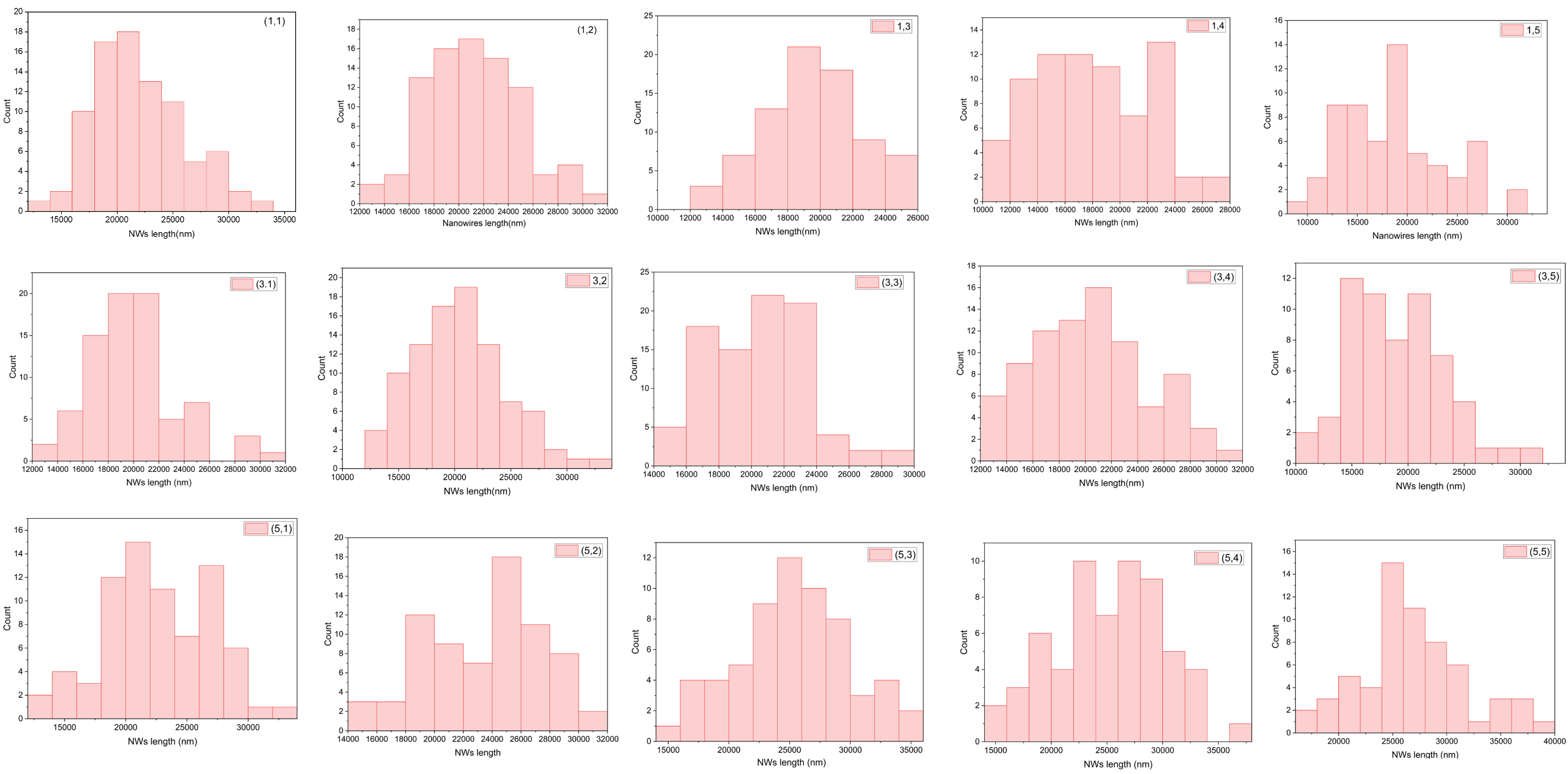


Fig.S3-1. Distribution of length of NWs in rows 1,3 and 5 of printed pattern.


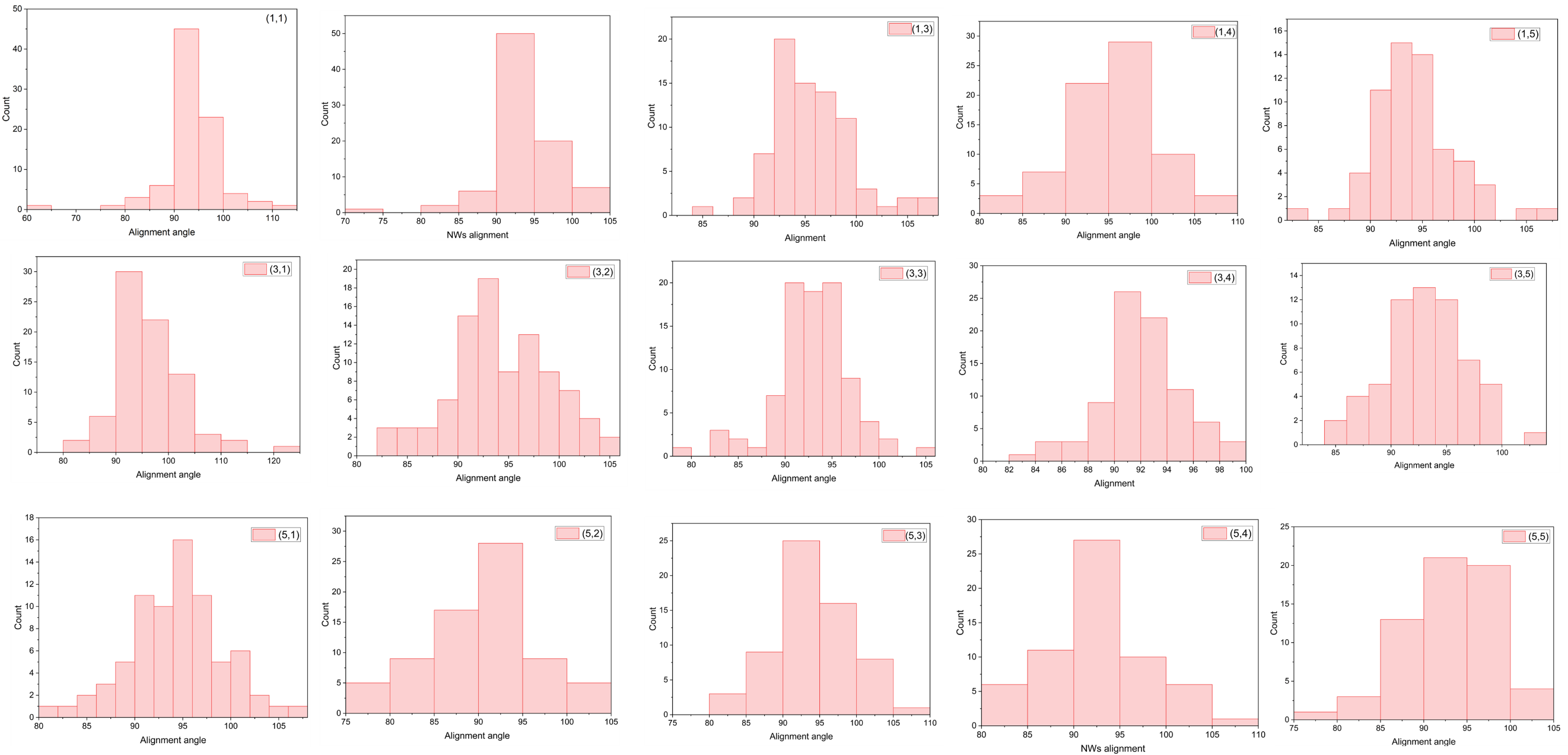


Fig.S3-2. Distribution of alignment of NWs in rows 1,3 and 5 of printed pattern.
